# Complete blood count parameters may have a role in diagnosis of gestational trophoblastic disease

**DOI:** 10.12669/pjms.313.7109

**Published:** 2015

**Authors:** Fatma Eskicioglu¹, Burcu Artunc Ulkumen, Esat Calik

**Affiliations:** 1 Fatma Eskicioglu, Celal Bayar University, School of Medicine, Department of Obstetrics and Gynecology, 45050 Manisa, Turkey; 2 Burcu Artunc Ulkumen, Celal Bayar University, School of Medicine, Department of Obstetrics and Gynecology, 45050 Manisa, Turkey; 3 Esat Calik, Celal Bayar University, School of Medicine, Department of Obstetrics and Gynecology, 45050 Manisa, Turkey

**Keywords:** Gestational trophoblastic disease, Platelet activation, Leukocyte count

## Abstract

**Objective:**

The goal of this study was to investigate whether gestational trophoblastic disease (GTD) and healthy pregnancy differ with respect to complete blood count parameters and these parameters can be used both to explain the pathophysiologic mechanisms and differentiate the two conditions from each other.

**Methods:**

The data obtained from 37 women with GTD and 61 healthy pregnancies (control group) regarding platelet (PLT), mean PLT volume (MPV) and PLT distribution width (PDW), and white blood cell (WBC) levels were evaluated. Patients with GTD were further subdivided into two groups composed of 20 partial mole (PM) and 17 complete mole (CM) cases.

**Results:**

PDW and WBC were lower in the GTD than the control. There were no differences for PLT and MPV. WBC was lower in PM and both WBC and PDW were lower in CM compared with control. ROC curve analysis revealed an area under curve (AUC) 75.5% for WBC and AUC 69.3% for PDW. A cut-off value was determined 8.19 for WBC with 81.0% sensitivity and 54.1% specificity. While, 15.85 were accepted for PDW, with 87.9% sensitivity and 44.4% specificity.

**Conclusion:**

Lower WBC in GTD may suggest that molar pregnancy requires a lower inflammatory reaction facilitating trophoblastic invasion. Lower PDW as an indicator of platelet activation in CM may suggest that CM requires less PLT activation than healthy pregnancy that needs stronger trophoblast invasion for normal placental development. Decreased PDW levels especially < 15.85 and WBC levels < 8.19 may alert clinicians for risk of GTD.

## INTRODUCTION

Gestational trophoblastic disease (GTD) is a tumor characterized by proliferation of trophoblasts originating from the placenta. It has a wide clinical spectrum consisting of partial (PM) and complete hydatidiform mole (CM), invasive mole, choriocarcinoma, and placental site trophoblastic tumor. Trophoblastic neoplasia (invasive mole or choriocarcinoma) occurs in 15-20% of CM and less than 5% of PM. Gestational trophoblastic neoplasia are potentially curable even in the presence of widespread metastatic disease. Treatment of GTD can only be provided with an accurate, early diagnosis and appropriate treatment.^1^

Complete hydatidiform mole mostly (80-90%) presents with vaginal bleeding. Other typical clinical signs and symptoms are uterine enlargement greater than expected for gestational age, hyperemesis, and pregnancy-induced hypertension in the first or second trimester.^2^ These signs and symptoms are often not observed in the partial mole. More than 90% of patients with partial mole have symptoms of incomplete or missed abortion, and the diagnosis is usually made after histological examination of curettage specimens.^3^ Beta human chorionic gonadotropin (beta - hCG) as a serum biochemical parameter is most commonly used in diagnosis and monitoring of GTD. Beta - hCG levels should be monitored as a surrogate marker for regression during disease and after treatment.^4^

Leukocytosis is a physiological finding during intrauterine healthy pregnancy.^5^ In addition, dilutional thrombocytopenia secondary to increased intravascular volume and compensatory increase in mean platelet volume (MPV) are also observed.^6^ MPV is a simple platelet (PLT) index and the combined use of MPV and PLT distribution width (PDW) is a more specific marker of PLT activation, which could more efficiently predict PLT activation.^7^ Increased MPV, PDW levels and leukocyte count are more prominent in preeclampsia that is characterized by an abnormal placental invasion and an exaggerated inflammatory response compared with healthy pregnancy.^6^,^8^ GTD can also lead to preeclampsia and hyperemesis gravidarum, another form of complicated pregnancy.^9^ However, there is a limited number of studies investigating differences in complete blood count (CBC) parameters in molar pregnancy and these studies have only focused on PLT count.^10^,^11^

To the best of our knowledge, there are no studies investigating the relation between molar pregnancy and PLT, MPV, PDW, and WBC. The main purpose of this study was to explore differences that allow differentiation of missed or incomplete abortion from GTD in which hCG levels are monitored. We also aimed to explain the pathophysiological mechanisms of molar pregnancies with the help of these parameters.

## METHODS

This study was carried out at the obstetric and gynecology department of a tertiary center. It was approved by Institutional Review Board. GTD (n = 37) patients (20 PM, 17 CM) diagnosed between 2004 and 2014 formed the study group. The control group consisted of first-trimester healthy pregnant (n = 61) women with ultrasonically confirmed fetal heart beat. These patients were assessed in terms of PLT, MPV, PDW, and WBC count as well as maternal demographic characteristics. The exclusion criteria were as follows: having a chronic disease including a chronic inflammatory disease, renal, cardiac or liver disease, pre-eclampsia; using drugs that affect coagulation cascade, including oral contraceptives, anticoagulants, and anti-inflammatory drugs; smoking; having hemoglobinopathy or coagulopathies.

GTD diagnosis was confirmed by pathology studies. The blood samples were obtained from GTH patients after admission to our clinic but before therapeutic interventions were contemplated. All blood samples were collected in EDTA (potassium ethylenediaminetetraacetate) containing tubes that served as an anticoagulant agent. Blood samples were analyzed within two hours after sampling with a commercially available analyzer (MINDRAY BC-6800).

### Statistical Analysis

The statistical package SPSS for Windows 15.0 (Statistical Package for Social Sciences; SPSS Inc., Chicago, IL) was used to analyze the data. Statistical comparisons between groups were performed using the Student’s *t* test and the Mann-Whitney U test. Mean and standard deviations were used to describe data. P values less than 0.05 were considered statistically significant. ROC analysis was performed to investigate the diagnostic performance of any marker.

## RESULTS

Demographic data is shown on Table-I. No statistically significant differences were observed between the groups with respect to age, number of previous pregnancies, deliveries, abortions, living children or gestational age.

**Table-I:** Demographic data of gestational trophoblastic disease (GTH) and control groups (mean±SD, Standard Deviation).

	GTH (n=37)	Control (n=61)	P value
Age	29.8±8.7	27.2±5.0	0.06
Gravida	2.6±1.4	2.2±1.3	0.17
Parity	1.1±1.2	1.0±1.2	0.46
Abortions	0.4±0.6	0.3±0.5	0.61
Living children	1.1±1.1	0.8±1.1	0.28
Gestational age (weeks)	7.9±1.5	7.9±1.9	0.85

**Fig.1:**
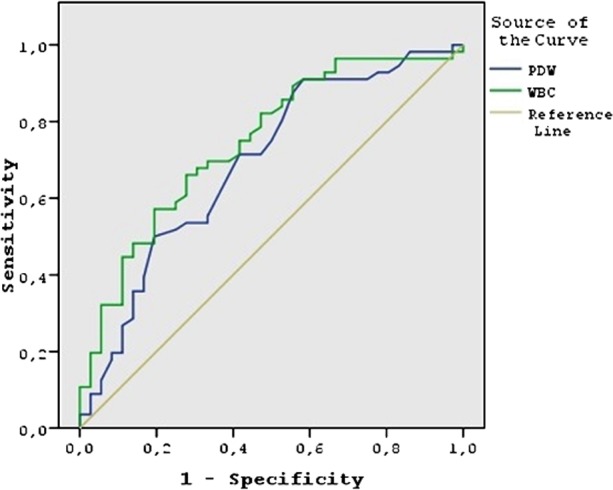
ROC analysis of WBC and PDW for GTD. AUC (area under curve) for WBC: 75.5%. AUC for PDW: 69.3%. ROC analysis indicates a cut-off value 8.19 for WBC with 81.0% sensitivity and 54.1% specificity and 15.85 for PDW cut-off value with 87.9% sensitivity and 44.4% specificity.

PLT, MPV, PDW, WBC levels of GTD (PM and CM) and control groups were shown on Table-II. There was no difference between GTD and control groups with respect to PLT and MPV levels. PDW and WBC levels were significantly lower in GTD group than the controls. When patients with GTD were further sub-classified into the PM and the CM groups, there were no significant differences between the PM and CM patients in terms of PLT, MPV, PDW, and WBC levels (Table-III). WBC count was significantly lower in the PM compared to the control group. WBC and PDW were significantly lower in CM than the control group (Table-III). ROC curve analysis for WBC revealed an area under curve (AUC) 75.5%. By using a cut-off value 8.19 for WBC, sensitivity was 81.0% and specificity was 54.1% for GTD. While, ROC curve analysis for PDW revealed an AUC 69.3%. By using a cut-off value 15.85 for PDW, sensitivity was 87.9% and specificity was 44.4% for GTD (Fig.1).

**Table-II:** Platelet (PLT), mean platelet volume (MPV), platelet distribution width (PDW), and white blood cell (WBC) levels in gestational trophoblastic disease (GTH) and control groups (mean±SD, Standard Deviation).

	GTH (n=37)	Control (n=61)	P value
PLT (10^3/µL)	242.6±77.5	221.3±65.1	0.14
MPV (fL)	9.1±1.3	9.7±1.7	0.07
PDW(fL)	15.8±1.8	16.7±1.2	0.004
WBC (10^3/µL)	8.5±2.9	11.8±4.0	<0.001

**Table-III:** Platelet (PLT), mean platelet volume (MPV), platelet distribution width (PDW), and white blood cell (WBC) levels in partial mole (PM) and complete mole (CM) subgroups (mean±SD, Standard Deviation).

	PLT(10^3/µL)	MPV(fL)	PDW(fL)	WBC(10^3/µL)
PM	239.10±69.45	9.29±1.25	16.01±2.16	7.69±2.21
CM	246.82±88.04	8.91±1.42	15.68±1.40	9.56±3.37
P value *	0.89	0.42	0.34	0.97
P value **	0.46	0.43	0.94	<0.001
P value ***	0.05	0.05	0.001	0.02

* PM versus CM group

** PM versus control group

*** CM versus control group.

## DISCUSSION

This is perhaps the first study that specifically explored the relationship between complete blood count (CBC) parameters and GTD. We found no significant differences between the groups with regard to PLT and MPV levels. However, GTD group had a significantly lower WBC level than the control group. PDW level was significantly lower in GTD than the controls, and this difference was largely driven by the patients with CM. ROC curve analysis for WBC and PDW levels revealed that these levels may be used for discriminating high risk population for GTD. By using a cut-off value 8.19 for WBC, sensitivity was 81.0% and specificity was 54.1% for GTD. By using a cut-off value 15.85 for PDW, sensitivity was 87.9% and specificity was 44.4% for GTD.

Molar pregnancies and gestational trophoblastic neoplasms are originated from placental trophoblasts. Normal trophoblasts include cytotrophoblasts, syncytiotrophoblasts, and intermediate trophoblasts. Syncytiotrophoblasts have the role of invasion of endometrial stroma to facilitate implantation of blastocyst. They also produce hCG. The role of cytotrophoblasts is to provide the syncytium with cells in addition to forming outpouchings that later become the chorionic villi covering the chorionic sac. The basal layer of the endometrium and the villous chorion adjacent to the endometrium form the functional placenta that is responsible for feto - maternal nutrient and waste substance exchange. Intermediate trophoblasts are located in the villi, the implantation site, and the chorionic sac. Hydatidiform mole is characterized by varying degrees of trophoblastic proliferation. Based on both morphologic and cytogenetic criteria, two syndromes of hydatidiform mole have been described: partial and complete.^12^,^13^

The pathophysiologic mechanism underlying molar pregnancies is still not clear. In CM, defective placentation due to lack of villous trophoblast development and endovascular trophoblastic invasion may lead to an incomplete development of the placenta - decidual interface. However, there is no reduction of normal endovascular decidual trophoblastic invasion in PM. Hence, it has been suggested that that PMs are commonly polypoid. Therefore, expression of additional maternal genetic component of PM different than that of CM, may be reason for sufficient interaction between trophoblasts and decidual layer.^14^

CBC is routinely used for examination of pregnant women. PLT, WBC, and PLT indices such as MPV and PDW are routinely studied in CBC samples. It is known that there is a physiological increase in WBC count during pregnancy.^5^ Leukocytosis is a result of an inflammatory process.^15^ In the beginning of pregnancy during the implantation phase various immunologic events take place, such as leukocyte activation and expression of various adhesion molecules on activated leucocytes.^16^ Large granular lymphocyte count is also elevated, which is thought to be originated from bone marrow in mid luteal phase in which implantation occurs. These cells are very active by secreting granulocyte / macrophage stimulating factor that helps trophoblastic invasion.^17^ We found that WBC count was lower in molar pregnancy than healthy pregnancy. The relation between WBC and GTD may be due to inadequate placentation in molar pregnancy, especially in CM, that result from absence of villous development and cytotrophoblastic invasiveness.^14^,^10^

CBC repeated during the course of a pregnancy exhibit a decreased PLT count during third trimester depending on the rise of plasma volume.^18^ PLT volume indicates PLT activation and increased production rather than PLT count.^6^,^19^ Soluble factors released from active PLTs increase the trophoblasts’ invasion capacity. By this way, they enable maternal spiral arteries to transform into low-resistance large-caliber veins.^20^ PDW and MPV are easily measured PLT indices that increase during PLT activation. PLTs change their shape to reach a larger surface during activation. It has been suggested that PLT activation causes an increase in both MPV and PDW resulting from PLT swelling and pseudopodia formation. PDW is a more specific marker of PLT activation since it does not increase during simple PLT swelling.^7^ In literature there are many studies aiming to relate elevated MPV and PDW to preeclampsia, the hypertensive disease of pregnancy.^6^,^8^,^19^ The idea that elevated MPV values are predictive of preeclampsia has been suggested by some studies.^6^ However, some studies have not supported that notion.^19^ The contradictory results have been suggested to arise from differences in measuring technology and substrates used for anticoagulation. In general, EDTA is used as the anticoagulant in blood count samples. When EDTA is used for anticoagulation instead of sodium citrate, MPV values tend to elevate depending on the elapsed time. EDTA - induced changes in PLT shape lead to a progressive increase in MPV.^6^,^19^ Both MPV and PDW are more effective in evaluating PLT activation.^7^ During intrauterine invasion of pregnancy, trophoblastic growth and differentiation are provided by trophoblasts while cytokines are released from endometrium and decidual stromal cells.^21^ However, CM is characterized by an incomplete development of placenta - decidual interface. Therefore, unchanged MPV values in contrast to lowered PDW level, a stronger sign of PLT activation, in molar pregnancies made us consider that less PLT activation is required in GTD (especially CM), a condition that is characterized with defective placentation and trophoblast invasion.^14^

PLT activation takes part in inflammatory reactions and immune responses through regulated expression of adhesion and immune receptors, release of inflammatory mediators and cytokines, and recruitment of leukocytes.^22^-^24^ Both decreased PDW and WBC in our study was supported by this interaction between the number of leukocytes and PLT activation. We aimed to study with a larger sample size in this study. However, as our study explored ten years’ data conducted at the tertiary center, our sample size remained small because of the low incidence of the disease.

## CONCLUSION

A significant difference in PDW value indicative of PLT activation in CM made us think that CM requires less PLT activation compared to healthy pregnancy requiring a stronger trophoblast invasion for normal placental development. Lower WBC levels compared to healthy pregnancy may suggest that molar pregnancy is associated with a poorer inflammatory function contributing trophoblastic invasion. These observations support the notion that defective placentation due to lack of trophoblast invasion has a role in etiology of in molar pregnancy. WBC levels <8.19 may predict GTD with 81.0% sensitivity and 54.1% specificity. While, PDW levels <15.85 may indicate GTD with 87.9% sensitivity and 44.4% specificity. Further studies with larger sample size should be conducted to use PDW and WBC levels for diagnosis of molar pregnancy.
